# Chronic and Latent Viral Infections and Leukocyte Telomere Length across the Lifespan of Female and Male Individuals Living with or without HIV

**DOI:** 10.3390/v16050755

**Published:** 2024-05-10

**Authors:** Nancy Yi Yang, Anthony Y. Y. Hsieh, Zhuo Chen, Amber R. Campbell, Izabella Gadawska, Fatima Kakkar, Laura Sauve, Ari Bitnun, Jason Brophy, Melanie C. M. Murray, Neora Pick, Mel Krajden, Hélène C. F. Côté, CIHR Team on Cellular Aging and HIV Comorbidities in Women and Children (CARMA)

**Affiliations:** 1 Department of Pathology and Laboratory Medicine, University of British Columbia, Vancouver, BC V6T 1Z7, Canada; nancyyang6@outlook.com (N.Y.Y.); anthony_y_hsieh@hotmail.com (A.Y.Y.H.); mel.krajden@bccdc.ca (M.K.); 2 Centre for Blood Research, University of British Columbia, Vancouver, BC V6T 1Z4, Canada; 3 Department of Microbiology and Immunology, University of British Columbia, Vancouver, BC V6T 2A1, Canada; 4 Women’s Health Research Institute, British Columbia Women’s Hospital and Health Centre, Vancouver, BC V6H 2N9, Canada; lsauve@cw.bc.ca (L.S.); melanie.murray@cw.bc.ca (M.C.M.M.);; 5 Oak Tree Clinic, BC Women’s Hospital and Health Centre, Vancouver, BC V5Z 0C9, Canada; 6 Department of Pediatrics, CHU Sainte-Justine, Université de Montréal, Montreal, QC H3T 1C5, Canada; fatima.kakkar@umontreal.ca; 7 Department of Pediatrics, University of British Columbia, Vancouver, BC V6H 3V4, Canada; 8 Department of Pediatrics, Hospital for Sick Children, University of Toronto, Toronto, ON M5G 1X8, Canada; ari.bitnun@sickkids.ca; 9 Department of Pediatrics, Children’s Hospital of Eastern Ontario, University of Ottawa, Ottawa, ON K1H 8L1, Canada; jbrophy@cheo.on.ca; 10 Department of Medicine, Division of Infectious Diseases, University of British Columbia Faculty of Medicine, Vancouver, BC V5Z 1M9, Canada; 11 British Columbia Center for Disease Control, Vancouver, BC V5Z 4R4, Canada

**Keywords:** herpesviruses, latent viruses, aging, telomere, HIV, sex differences, virology, hepatitis

## Abstract

Background: Chronic/latent viral infections may accelerate immunological aging, particularly among people living with HIV (PLWH). We characterized chronic/latent virus infections across their lifespan and investigated their associations with leukocyte telomere length (LTL). Methods: Participants enrolled in the CARMA cohort study were randomly selected to include n = 15 for each decade of age between 0 and >60 y, for each sex, and each HIV status. Cytomegalovirus (CMV), Epstein–Barr virus (EBV), human herpesvirus 8 (HHV-8), herpes simplex virus 1 (HSV-1), and HSV-2 infection were determined serologically; HIV, hepatitis C (HCV), and hepatitis B (HBV) were self-reported. LTLs were measured using monochrome multiplex qPCR. Associations between the number of viruses, LTL, and sociodemographic factors were assessed using ordinal logistic and linear regression modeling. Results: The study included 187 PLWH (105 female/82 male) and 190 HIV-negative participants (105 female/84 male), ranging in age from 0.7 to 76.1 years. Living with HIV, being older, and being female were associated with harbouring a greater number of chronic/latent non-HIV viruses. Having more infections was in turn bivariately associated with a shorter LTL. In multivariable analyses, older age, living with HIV, and the female sex remained independently associated with having more infections, while having 3–4 viruses (vs. 0–2) was associated with a shorter LTL. Conclusions: Our results suggest that persistent viral infections are more prevalent in PLWH and females, and that these may contribute to immunological aging. Whether this is associated with comorbidities later in life remains an important question.

## 1. Introduction

Life expectancy has been increasing for people living with HIV (PLWH) who can access effective combination antiretroviral therapy (cART) [1]. However, despite the benefits of cART, PLWH are still at a higher risk of experiencing age-related comorbidities earlier in life [2], including cardiovascular disease [3,4,5,6,7], liver disease [8], bone disease [2,9,10], and neurocognitive impairments [11,12,13,14].

In line with the earlier onset of age-associated diseases in PLWH, biomarkers of biological aging have also been linked to HIV infection [15]. One such marker is the length of telomeres, as their shortening is one of the hallmarks of biological aging [16,17]. Several studies have reported shorter telomere lengths in the blood cells of PLWH [18,19], although many other factors such as male sex, certain ethnicities, social determinants of health, and tobacco smoking are also associated with shorter telomeres [16,20,21,22,23,24].

It is well established that the ability to mount strong immune responses declines with age. Chronic and latent viral infections such as HIV, hepatitis C virus (HCV), or cytomegalovirus (CMV) elicit chronic and/or repeated immune activation, and as such represent stressors that contribute to immune aging and senescence. Many chronic and latent viral infections have been shown to be more prevalent amongst PLWH (Table 1). These include members of the herpesvirus family such as CMV, herpes simplex 1 and 2 viruses (HSV-1, HSV-2), Epstein–Barr virus (EBV), and human herpesvirus 8 (HHV-8), as well as hepatitis B virus (HBV) and hepatitis C virus (HCV). Apart from HCV, which can now be eradicated with antiviral therapy, these viral infections are usually lifelong and can either be chronic (HIV, HBV, HCV) or latent with periodic reactivations (HSV-1, HSV-2, CMV, EBV, HHV-8). Whether the infection is acute, chronic, or reactivated from latency, these viruses trigger immune activation and inflammation, promoting the proliferation of immune cells which lead to immune senescence. Chronic inflammation and cellular senescence are hallmarks of biological aging, and these viruses have all been epidemiologically linked with age-associated non-communicable diseases and/or cancers, although the mechanisms behind these associations are often unclear. Most of these persistent viruses have also been associated with a shorter telomere length in immune cells, as summarized in Table 1. Although several studies have investigated the effect of persistent viruses on markers of immune aging, few have examined multiple viruses at once, and over the lifespan of a person. Understanding their cumulative immunological effect could help guide prevention and/or treatment strategies, especially for PLWH.

In this cross-sectional study, we sought to determine the seroprevalence of seven latent/chronic infections (HSV-1, HSV-2, EBV, CMV, HHV-8, HBV, and HCV) in a sample of male and female study participants living with HIV and not, distributed across the human lifespan, from <1 to 76 years. Given that harboring multiple chronic viruses and/or latent viruses can contribute to immunological aging, our aim was to also investigate the effect of sex and HIV status as predictors of viral infections and examine the associations between viruses and leukocyte telomere length (LTL).

## 2. Materials and Methods

### 2.1. Study Design and Study Participants

This study is a cross-sectional nested case–control observational study. It was designed to include participants who are well-balanced with respect to sex, HIV status, and age, and to include approximately 15 individuals in each decade of life from age 0 to 60+ for each HIV status and sex (Table 2).

Study participants were enrolled in the CARMA (Children and women: AntiRetroviral therapy and Markers of Aging) cohort study. Adult men and women living with HIV as well as HIV-negative controls with similar sociodemographic characteristics were enrolled in Vancouver, British Columbia, from 2008 to 2018. Additional adult participants were later recruited between 2020 and 2022 in Vancouver, through online and poster advertisements, in an effort to reach the target of 15 individuals per age group (Table 2). The COVID-19 pandemic hindered recruitment, and thus we did not reach the target number in all age groups. 

Between 2008 and 2017, children (1 month to 19 years) were enrolled in the CARMA cohort at four sites across Canada: British Columbia Women’s Hospital in Vancouver, British Columbia; the Centre Hospitalier Universitaire Sainte-Justine in Montreal, Quebec; the Hospital for Sick Children in Toronto, Ontario; and the Children’s Hospital of Eastern Ontario in Ottawa, Ontario. The children were either living with HIV or born to mothers living with HIV and herein considered as part of the HIV-negative group, although they would also be referred in the literature as children HIV-exposed but uninfected (CHEU). Study visits took place annually between 2008 and 2013, and then every 2–3 years thereafter. The inclusion criteria for CARMA were to be living with HIV, or not, and able to provide informed consent/assent.

### 2.2. Biospecimen Collection and Serology

Peripheral venous whole blood was collected from participants in British Columbia, Ontario, and Quebec and was either stored overnight at room temperature (British Columbia) or shipped overnight to Vancouver (Ontario, Quebec), before being stored at −80 °C. Infection status was determined by commercial ELISA of serum and/or plasma biospecimens, depending on specimen availability. For HSV-1 and HSV-2, serology was obtained at the British Columbia Centre for Disease Control, using in-house ELISA. The testing for EBV (Calbiotech EV010G EBV-VCA IgG, San Diego, CA, USA), CMV (Calbiotech CM027G CMV IgG, San Diego, CA, USA), and HHV-8 (Abbexxa ABX157142 HHV-8, Cambridge, UK) was carried out using the cited commercial ELISAs, according to the manufacturer’s protocols. Concordance between serum and plasma serology was determined and the results are shown in Table S1. 

### 2.3. DNA Extraction and LTL Quantification

Frozen whole blood was thawed and genomic DNA was extracted from 100 µL using the QIAamp DNA Mini Kit with the QIAcube (Qiagen, Hilden, Germany), according to the manufacturer’s blood and body fluid protocol. Extracted DNA was eluted in 100 µL of kit buffer AE and stored at −80 °C until assayed for telomere length. LTL is a relative measure of telomere length defined as the ratio (T/S) between the quantities of telomeric DNA and albumin (ALB), a single-copy nuclear gene. The relative LTL was quantified using a previously described monochrome multiplex real-time quantitative PCR (MMqPCR) assay using the LightCycler 480 [57].

### 2.4. Self-Reported Data

Demographic data were self-reported during structured questionnaires administered by trained research staff during the study visits. Two data collection forms were used: a “pediatric” form for participants under the age of 16 and another form for participants over the age of 16. HCV and HBV infection history were self-reported and confirmed by medical records when possible. For a few participants (n = 5) whose HCV and/or HBV status was recorded as “unknown” or “not asked”, usually pediatric participants, the data were imputed as “never infected”.

### 2.5. Statistical Analyses

The two primary measures of interest were the number of chronic viral infections present and LTL. To investigate the relationships between these and our variables of interest, namely age, sex, and HIV status, we first carried out bivariate analyses for each measure using Pearson’s or Spearman’s correlation, Mann–Whitney U, or unpaired Student’s *t*-tests depending on data distribution and continuous/categorical nature of the variable. Variables were selected based on these and a priori decisions for inclusion in our multivariable modeling by linear or logistic regression. In addition to age, sex, and HIV status, the variables considered for inclusion in the model also included tobacco smoking, ethnicity, and region of birth. The final multivariable models were constructed in order to maximize statistical power by decreasing the Akaike’s Information Criterion and increasing the R-squared. Variance inflation factors were used to estimate the collinearity among variables, and interaction terms were included in the model if an interaction was detected. Analyses were repeated among HIV groups or those segregated by sex. Statistical analyses were performed using JMP Pro version 16.0.0.

## 3. Results

The study participants’ characteristics are presented in Table 3 and Table 4. Their region of birth is shown in Table S2.

### 3.1. Higher Prevalence of Viruses among Participants with HIV and of Female Sex

**Figure 1:**
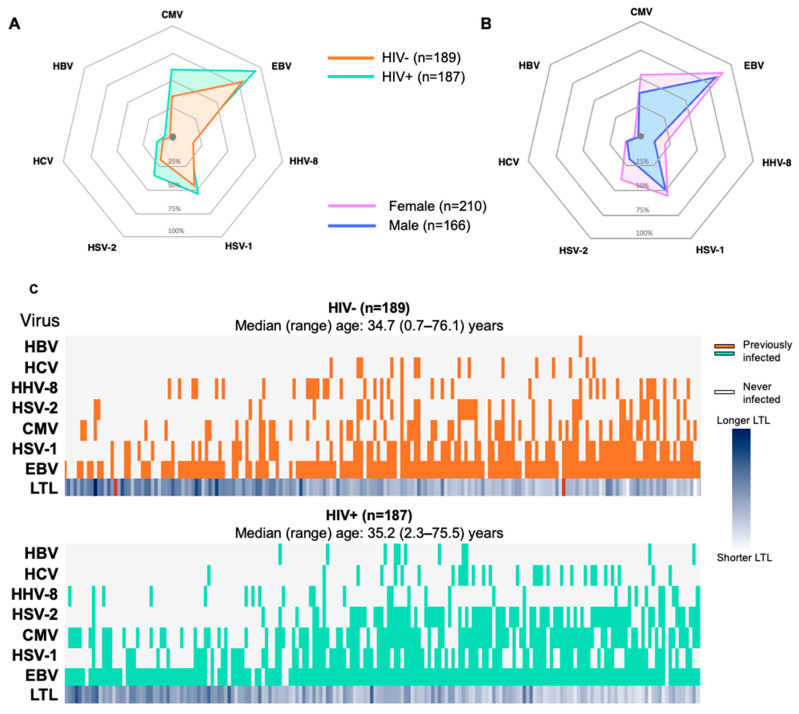
Prevalence of chronic/latent viral infections. Radar plots depicting the percentage of participants who have ever been infected with each of the following chronic/latent viruses: hepatitis B virus (HBV), hepatitis C virus (HCV), human herpes virus 8 (HHV-8), herpes simplex virus 2 (HSV-2), cytomegalovirus (CMV), herpes simplex virus 1 (HSV-1), and Epstein–Barr virus (EBV), segregated by HIV status (**A**) or sex (**B**). Panel (**C**) shows a heat map depicting the presence (coloured) or absence (blank) of each virus amongst all participants, with age increasing from left to right, for the HIV-negative group (orange, top panel) and the group living with HIV (bottom panel, green). Rows depict virus type, and each column depicts a distinct participant. The median (range) age is indicated for each group.

As presented in Figure 1A, all of the non-HIV chronic/latent viruses studied, except HHV-8, were more prevalent among PLWH than in the HIV-negative controls. A similar pattern was also observed whereby a slightly higher percentage of female participants were infected with each of the seven non-HIV viruses studied relative to their male counterparts (Figure 1B, Table S3). As shown in Figure 1C, viruses such as CMV, EBV, and HSV-1 often appear at a young age, while other infections such as HSV-2 and HCV were acquired at older ages. The last row in both heat maps represents the LTL per participant (darker shades representing longer LTLs). There appears to be a trend of shorter LTLs with older age, as expected.

### 3.2. LTL Decreases with Age in Both Female and Male Participants

**Figure 2:**
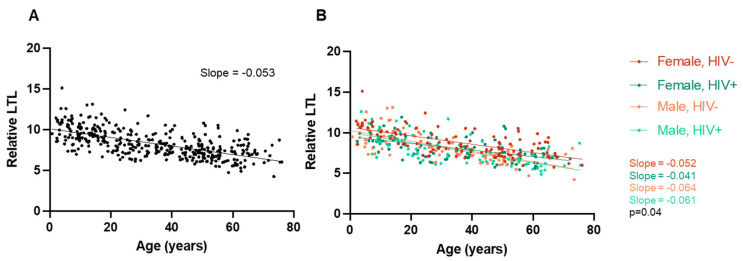
(**A**) Relative LTL decreases with older age. Scatterplot depicting all participants, where older age is correlated with shorter LTLs (n = 374, Pearson’s r = −0.64, *p* < 0.0001). (**B**) Same relative LTL data segregated by subgroup, namely the female HIV- group (n = 104, r = −0.65, *p* < 0.0001), female HIV+ group (n = 105, r = −0.58, *p* < 0.0001), male HIV- group (n = 83, r = −0.71, *p* < 0.0001), and male HIV+ group (n = 82, r = −0.71, *p* < 0.0001). The differences between the slopes of the four different regression lines are significantly different (*p* = 0.04).

Amongst all participants, in the sex-segregated and HIV-segregated groups, we confirmed that relative LTL significantly decreases with advancing age (Figure 2). This result was expected and provides a baseline for the interpretation of further results.

**Figure 3:**
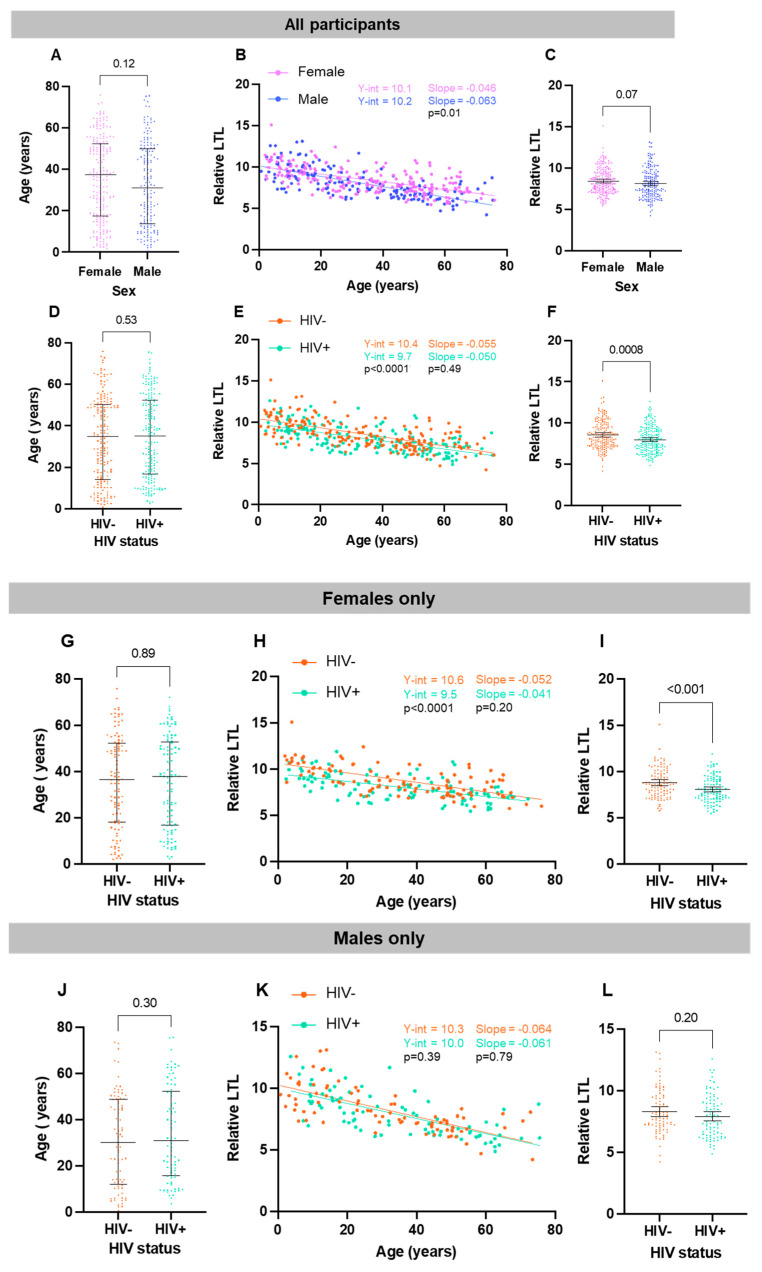
Relative LTL significantly decreases with older age in both female and male groups. (**A**) There is no significant difference in age between all male (n = 165) and all female participants (n = 209) (*p* = 0.12, Mann–Whitney test, median + IQR shown). (**B**) Scatterplot depicting all participants separated by sex. Increasing age is significantly associated with a shorter LTL amongst both female participants (Pearson r= −0.59, Pearson’s *p* < 0.0001) and male participants (r= −0.71, *p* < 0.0001). There is a significant difference in the slope of their regression lines, whereby LTL decreases at a steeper slope in male participants compared to female ones (*p* = 0.01). (**C**) There is no significant difference in LTL between male and female participants (*p* = 0.07, unpaired *t*-test, mean + 95% CI shown). Relative LTL significantly decreases with older age in both HIV+ and HIV- groups. (**D**) There is no significant difference in age between all HIV- (n = 187) and HIV+ (n = 187) participants (*p* = 0.53, Mann–Whitney test, median + IQR shown). (**E**) Scatterplot depicting all participants separated by HIV status. Increasing age is associated with a shorter LTL amongst both HIV- participants (r= −0.65, *p* < 0.0001) and HIV+ participants (r= −0.64, *p* < 0.0001). There is a significant difference in y-intercept (*p* < 0.0001) but not regression line slope (*p* = 0.49). (**F**) HIV+ participants have significantly shorter LTLs compared to HIV- participants (*p* = 0.008, unpaired *t*-test, mean + 95% CI shown). Relative LTL significantly decreases with older age in both HIV+ and HIV- female groups. (**G**) There is no significant difference in age between female HIV- (n = 104) and female HIV+ (n = 105) participants (*p* = 0.71, Mann–Whitney test, median + IQR shown). (**H**) Scatterplot depicting all female participants separated by HIV status. Increasing age is associated with a shorter LTL amongst both female HIV- participants (slope = −0.052, Pearson’s *p* < 0.0001) and female HIV+ participants (slope = −0.041, *p* < 0.0001). There is a significant difference in y-intercept (*p* < 0.0001) but not regression line slope (*p* = 0.20). (**I**) HIV+ female participants have significantly shorter LTLs compared to HIV- female participants (*p* = 0.0004, unpaired *t*-test, mean + 95% CI shown). Relative LTL significantly decreases with older age in both HIV+ and HIV- male groups. (**J**) There is no significant difference in age between male HIV- (n = 83) and male HIV+ (n = 82) participants (*p* = 0.30, Mann–Whitney test, median + IQR shown). (**K**) Scatterplot depicting all male participants separated by HIV status. Increasing age is associated with a shorter LTL amongst both male HIV- participants (slope = −0.064, *p* < 0.0001) and male HIV+ participants (slope = −0.061, *p* < 0.0001). There is no significant difference in y-intercept (*p* = 0.39) or regression line slope (*p* = 0.79). (**L**) There is no significant difference in LTL between male HIV- and male HIV+ participants (*p* = 0.20, unpaired *t*-test, mean + 95% CI shown).

We first confirmed that there were no differences in age between the groups according to sex (Figure 3A). For participants of both sexes, their relative LTL decreased significantly with increasing age (female: r = −0.59, *p* < 0.0001; male: r = −0.71, *p* < 0.0001), and this decline appeared faster among males, based on the difference between the slopes of the linear regressions (*p* = 0.01, Figure 3B). However, the difference in LTL between female and male participants did not reach statistical significance (8.4 [8.2–8.6] vs. 8.1 [7.8–8.4], *p* = 0.07) (Figure 3C).

We then made the same triad of comparisons, this time according to HIV status, and confirmed no significant difference in age between participants living with HIV and without HIV (Figure 3D). Again, in both the HIV-negative (r = −0.65, *p* < 0.0001) and PLWH (r = −0.64, *p* < 0.0001) groups, their LTL significantly decreased with increasing age and at a similar rate to one another (Figure 3E). However, despite being of similar ages, PLWH had significantly shorter LTLs compared to HIV-negative participants, as illustrated by both a significantly lower Y intercept (Figure 3E) and mean LTL (8.0 [7.8–8.2] vs. 8.6 [8.3–8.8], *p* = 0.0008) (Figure 3F).

### 3.3. HIV Is Associated with LTL in Female Participants

Given our results with respect to the prevalence of chronic/latent viral infections and relative LTLs, we next examined the relationships between LTL and HIV status within participants of each sex. First, we confirmed that there was no difference in age between the PLWH and HIV-negative groups for either sex (Figure 3G,J). Among the female participants, LTLs declined at similar rates with age in the two HIV groups but showed a lower y-intercept for the HIV group (Figure 3H). Among females, the LTL was significantly shorter in the group living with HIV vs. the control group (8.1 [7.8–8.3] vs. 8.8 [8.5–9.1]; *p* < 0.001) (Figure 3I). Among males, LTLs significantly decreased with increasing age in both groups (Figure 3J,K), but, in contrast to the results obtained in the female group, there was no significant difference in LTL between the group living with HIV vs. the negative controls (7.9 (7.5–8.3) vs. 8.3 (7.9–8.7, *p* = 0.20) (Figure 3L). This suggested an HIV*sex interaction whereby HIV affects LTL in female participants only. 

### 3.4. A Greater Number of Viruses Is Associated with Older Age, HIV Status, and Female Sex

**Figure 4:**
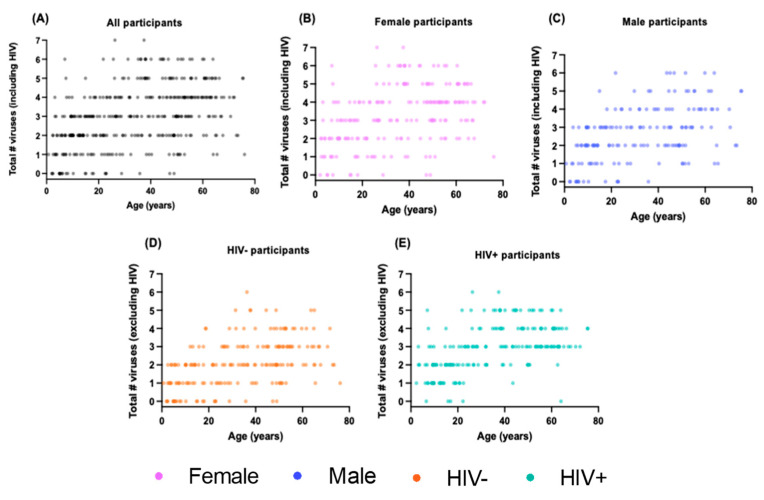
The total number of viral infections increases with age in all groups. Scatterplot depicting that older age is significantly correlated with having more viruses among (**A**) all participants (n = 376, Spearman’s rho = 0.45, *p* < 0.0001) and amongst (**B**) only female participants (n = 210, rho = 0.40, *p* < 0.0001), (**C**) only male participants (n = 166, rho = 0.48, *p* < 0.0001), (**D**) only HIV-negative participants (n = 189, rho = 0.51, *p* < 0.0001), and (**E**) only PLWH (n = 187, rho = 0.55, *p* < 0.0001).

**Figure 5:**
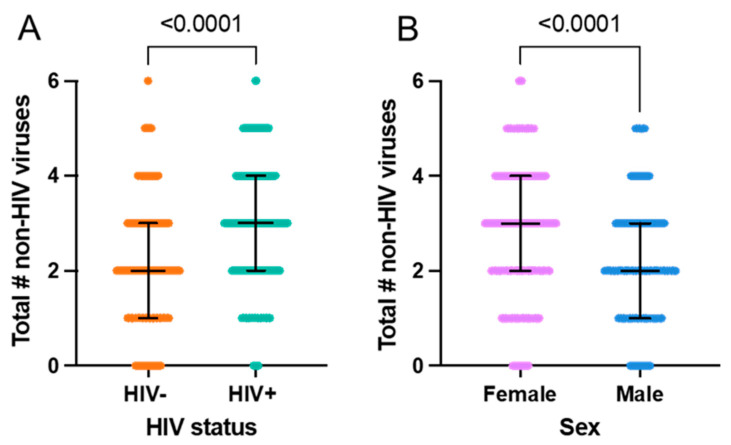
Number of non-HIV viruses according to HIV status and sex. (**A**) HIV+ participants have a significantly greater number of other viral infections compared to HIV- participants. Univariately, the HIV+ group has a greater number of non-HIV viruses (*p* < 0.0001); the median number of viruses is 2 amongst HIV- participants (n = 189) and 3 amongst HIV+ participants (n = 187). Median + IQR shown; Mann–Whitney test. (**B**) Female participants have a significantly greater number of non-HIV viral infections compared to male participants. Univariately, the female group has a greater number of non-HIV viruses (*p* < 0.0001); the median number of viruses is 3 for the female group (n = 210) and 2 for the male group (n = 166). Median + IQR shown; Mann–Whitney test.

As might be expected, the total number of chronic/latent viral infections increases with age in all groups examined (Figure 4), although several female participants stand out as having a large number of viruses given their age. Amongst PWLH, only two participants over the age of 30 harbored ≤ 1 non-HIV virus, compared to 16 people in the HIV-negative group (Figure 4D,E). There were no male participants but several female participants living with more than five non-HIV viruses. Despite being of similar age, PLWH live with a higher number of non-HIV viruses than HIV-negative participants (Figure 5A, median 3 vs. 2, *p* < 0.0001). In addition, as hinted at by the data shown in Figure 4, the female participants in the study have significantly more non-HIV viruses than male participants (Figure 5B, median 3 vs. 2, *p* < 0.0001).

### 3.5. Shorter LTL Associated with Having More Viral Infections

**Figure 6:**
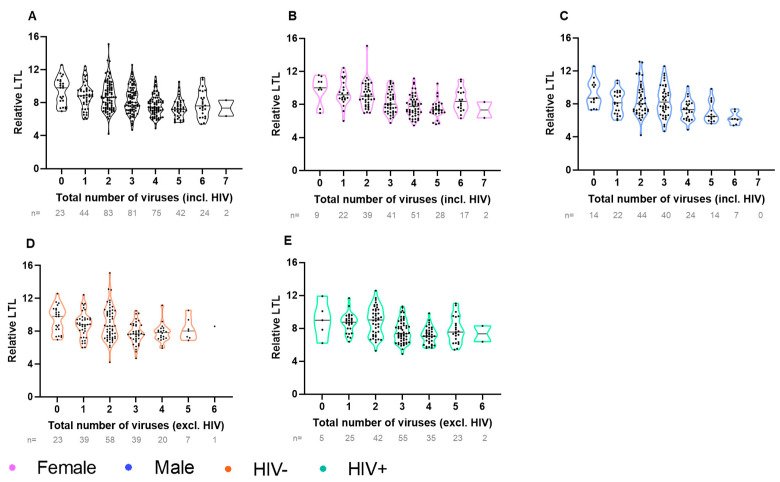
LTL decreases as the total number of viral infections increase. Violin plot depicting relative LTL univariately decreasing as the total number of viral infections increases in (**A**) all participants (n = 374, Spearman’s rho = −0.36, *p* < 0.0001), and amongst (**B**) only female participants (n = 209, rho = −0.40, *p* < 0.0001), (**C**) only male participants (n = 165, rho = −0.37, *p* < 0.0001), (**D**) only HIV- participants (n = 187, Spearman’s rho = −0.31, *p* < 0.0001), and (**E**) only PLWH (n = 187, Spearman’s rho = −0.34, *p* < 0.0001).

We next examined the relationship between the number of viruses and LTL. Among all four subgroups (female, male, HIV-negative, living with HIV), a higher number of chronic/latent viral infections was associated with a shorter LTL (Figure 6). The majority (69%) of participants harbored between one and three non-HIV viruses (Figure 6D,E).

### 3.6. Factors Independently Associated with the Number of Viruses

**Figure 7:**
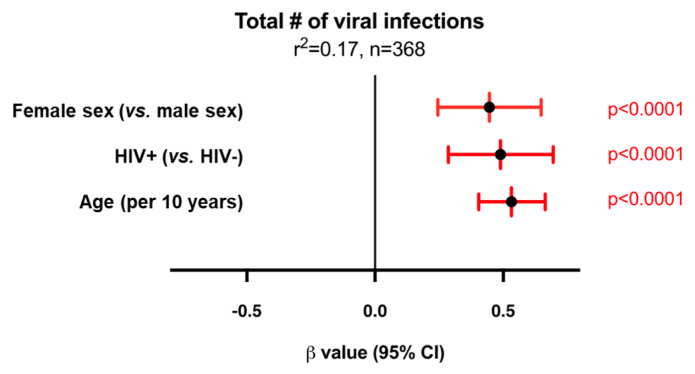
Multivariable logistic regression modelling of the total number of viral infections amongst all participants. The model shows that female sex, HIV+ status, and older age are independently associated with having more chronic/latent viral infections (after additional adjustment for ethnicity, tobacco smoking status, and region of birth). This model was selected by minimizing Akaike’s Information Criterion (AIC) and maximizing the coefficients of determination (r^2^). The center points depict the β value and lines depict 95% confidence internals. Red intervals designate statistical significance. Negative β values indicate that the specified variable has the fewer viral infections compared to the reference group, whereas positive β values indicate the presence of more viral infections. The coefficient of determination is shown at the top. n = 6 excluded due to missing data (region of birth); n = 2 excluded due to South American region of birth (too few events).

In our logistic regression model, older age, HIV infection, and female sex remained independently associated with the number of viral infections after adjusting for ethnicity, tobacco smoking, and region of birth (Figure 7, Table S4). Given the sex association observed, we carried out sex-disaggregated analyses (Figure S1). The independent association with HIV and older age remained for both sexes; however, other predictors behaved differently according to sex. For example, region of birth (Africa), and Indigenous ethnicity were associated with having a greater number of viruses among female participants only, with little or no evidence of this among male participants.

### 3.7. Factors Independently Associated with LTL

**Figure 8:**
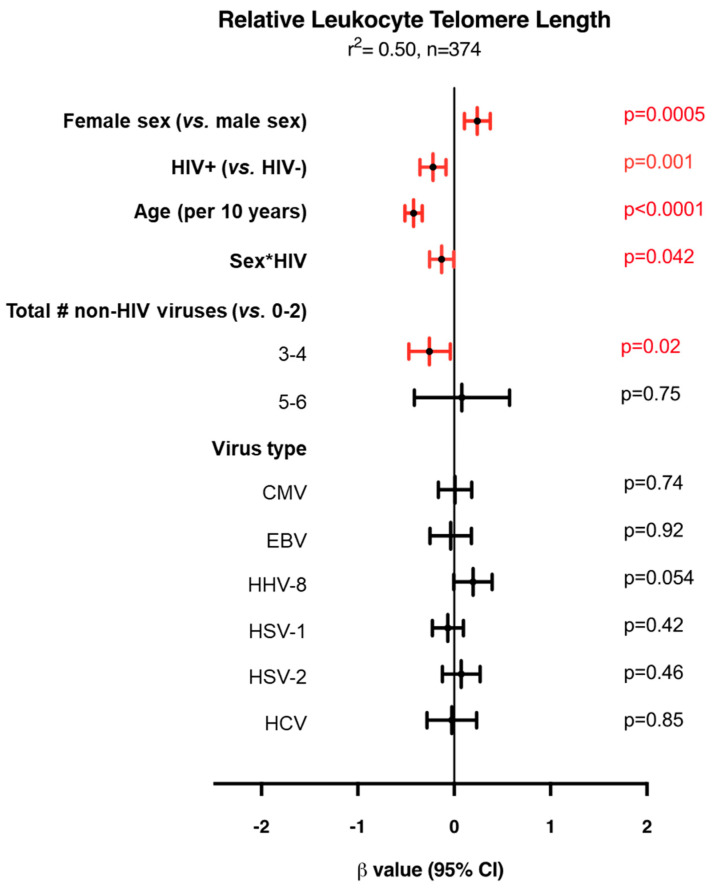
Multivariable linear regression modelling of relative leukocyte telomere length (LTL) amongst all participants. The model shows that after adjusting for sex, HIV status, age, Sex*HIV, and virus type, having 3–4 non-HIV viruses compared to 0–2 is independently associated with a shorter LTL (model also adjusted for ethnicity and tobacco smoking status). This model was selected automatically by minimizing Akaike’s Information Criterion (AIC). The center points depict the β value and the lines depict the 95% confidence internals. Red intervals designate statistical significance. Negative β values indicate that the specified variable has a shorter LTL compared to the reference group (vs), whereas positive β values indicate longer LTLs compared to the reference group. The coefficient of determination (r^2^) is shown at the top. n = 2 excluded due to missing data.

Amongst all participants, a multivariable linear regression model for LTL showed that older age, male sex, and HIV+ status are associated with shorter LTLs (Figure 8, Table S5). The association between sex and LTL suggested in Figure 3C becomes significant after adjusting for other variables. With respect to the relationship between the number of viruses and LTL, compared to living with zero, one, or two non-HIV viruses, having three or four of the seven non-viruses was associated with a significantly shorter LTL (*p* = 0.02). Of note, having five or more viruses did not show a significant association with LTL, although, due to the smaller numbers, the confidence interval was large, hence the estimate is less precise (Figure 8). However, a significant interaction between sex and HIV status was detected, whereby an association was seen between shorter LTLs and HIV status amongst female participants, but not male participants (Figure 3I,L). Not considering HBV due to small numbers, none of the six chronic/latent viruses were found to be independently associated with LTL, although HHV-8 infection tended toward an association with longer LTLs (*p* = 0.054).

## 4. Discussion

This is the first human cohort study to investigate the impact of chronic/latent viruses and the number of infections on a marker of immunological aging amongst people living with and without HIV. Apart from confirming that viruses accumulate with age, our main finding is that PLWH have more chronic viral infections, and that female participants harbor a significantly higher number of viruses than their male counterparts. This observation remained after adjusting for age, region of birth, and ethnicity. Our data further suggest that having a greater number of non-HIV viruses may also be associated with a shorter LTL, although the lack of significant association seen with five or more non-HIV viruses points to the need to confirm this observation in an independent cohort.

The finding that the female sex is associated with having more viruses may be related to differences in male and female susceptibility to virus acquisition and transmission. For example, HSV-2 is known to have a higher prevalence amongst females, as it is more transmissible from male to female during heterosexual intercourse [58,59]. Sex-specific models reveal that some predictors of chronic/latent infections are shared between sexes, for example older age and HIV, while others appear to clearly diverge, among them ethnicity and country of birth. These differences are unlikely explained by the differences in demographics, since both male and female participants shared similar demographic characteristics. However, this observation could reflect differences related to other factors such as the changes in immune tolerance experienced during pregnancy to not reject the fetus [60]. In addition, differences or changes in sex hormones likely influence virus acquisition and/or latent virus reactivation. For example, the transmission of genital tract infections (such as herpes viruses) increases with oral contraceptive use [61,62]. Other factors, possibly cultural or gender-role related, may partially explain these associations with demographic characteristics. Though this study was not designed to address those questions, our findings pave the way for future studies to investigate sex and related differences that could impact people’s health differently, and possible sex-specific prevention, screening, and/or treatment approaches.

Another factor associated with a greater number of viruses in both sexes was being a current smoker. This could be related to the depressive effect of tobacco smoking on the immune system [63] or increased exposure through the act of regularly exposing the mouth mucosa to the external environment, although past smoking did not show an association, as would be expected if this were the case. The association between an African region of birth and having more viruses is consistent with the literature stating that several of these viruses are highly endemic in parts of the world, such as sub-Saharan Africa (CMV, HSV-2, and HHV-8), and often acquired early in life [31,64]. It should be noted that this study utilized a pan-Canadian cohort with the majority of participants being born in North America (Table S2). Future studies on markers of aging should performed in populations where HIV seropositivity is higher and where other viral infections such as HHV-8 are endemic.

Overall, the associations seen between LTL and female sex, HIV status, age, and smoking are all consistent with previous knowledge. It is noteworthy that our model explained 50% of the LTL variance, which is higher than previous models [19]. Having 3–4 viruses being associated with a shorter LTL (compared to 0–2) can be explained by the mechanism of the protective function of telomeres. As the immune system exerts itself in the form of leukocyte replication to fight pathogens, so do the telomeres shorten. Therefore, accumulating more viruses can lead to greater immune senescence. The lack of a similar effect when one is infected with 5–6 viruses warrants further investigation; a possible explanation is that LTL loss is driven by specific viruses, such as those more likely to reactivate during one’s lifespan. The result of LTL decline with age occurring at a similar slope between PLWH and HIV-negative controls but at a lower y-intercept in the HIV group is consistent with our previous study showing that HIV acquisition appears to be accompanied by a rapid decline in LTL that then persists with time [65]. The outcome seen of longer average LTLs in females despite them having more overall viral infections is also worth noting. This could be due to sex differences with respect to immune regulation and inflammatory responses, with previous studies documenting such discrepancies and citing the mechanisms of genetics and sex-specific steroids [64,66,67,68]. To address potential sex-differentiated immune senescence, future studies should be designed to incorporate the quantification of inflammatory markers. The close-to-significant association seen between HHV-8 and longer LTLs is notable as it stands as the only virus in our multivariable model with an almost-independent association with LTL. One possible mechanism lies in the interaction between latency-associated nuclear antigen (LANA), an HHV-8 encoded antigen expressed by infected cells, and the enzymatic subunit of human telomerase reverse transcriptase (hTERT). One group proposed that LANA up-regulates hTERT promoter activity through a direct interaction with the Sp1 binding motifs located in the hTERT promoter sequence [69]. HHV-8 is not the only member of the herpes virus family to have been associated with elongated telomeres; previous studies have shown that human herpesvirus 6A/6B is able to integrate into telomeres due to its sequence homology [70], and other groups have shown that EBV can also enhance telomerase activity [71,72], warranting further investigation, given the lack of a similar effect in our model.

There are limitations in this study with respect to the lack of differentiation between active and cleared HCV infections. In this study, we investigated how past infection at any time may contribute to aging. However, chronic viruses such as HCV can be cleared following the initial infection spontaneously or with anti-viral treatment, which would impact participants’ levels of senescence. Future studies should utilize PCR methods to account for active HCV infections. Another limitation is being unable to account for the number of reactivations undergone by latent viruses at the time of the study visit. When a reactivation occurs, an immune response is mounted to fight the actively replicating pathogen, causing the proliferation and activation of leukocytes. A greater number of reactivations would therefore lead to greater immune exhaustion. To address this, future studies should aim to detect the number of reactivations undergone, potentially utilizing PCR methods to quantify the amount of viral genome in latently infected cells. We also could not investigate various factors such as poverty, crowded living conditions, family size, etc., which may play a role in the risk of virus acquisition, particularly herpesviruses. Lastly, there exist some limitations due to self-reported HCV, HBV, and HIV status. Overall, due to the known low prevalence of these viruses and previously conducted high concordance studies by our group, we expect the main findings to be unchanged if chronic infection status were serologically confirmed. 

In conclusion, we found that female sex, HIV infection, and specific sociodemographic factors can all play a role in increasing the number of chronic/latent viruses that can be accumulated during one’s lifetime. Furthermore, having more of these seemingly harmless viruses can have the effect of contributing to accelerated immunological aging.

## Figures and Tables

**Table 1:** Summary of major characteristics of seven chronic/latent viruses of interest.

Virus	Estimated Prevalence	Linked to Shorter TL in Immune Cells	Associated Age- Related Diseases	Main Modes of Transmission	Chronic vs. Latent Virus	References
CMV	Children: 20–70% HIV+ adults: >84% HIV- adults: 50–85%	Yes	Atherosclerosis, autoimmune disease; also associated with increased immune activation and inflammation in PLWH	Bodily fluids, perinatal	Latent. Infects a broad range of human cell types and is asymptomatic in most healthy individuals	[25,26,27,28,29,30,31,32,33]
EBV	Children: 54–83% HIV+ adults: ~90% HIV- adults: ~48%	Yes	Lymphoma (Burkitt’s, Hodgkin’s, and non-Hodgkin), gastric carcinoma, Multiple Sclerosis, Alzheimer’s disease	Bodily fluids, especially saliva	Latent. Acquisition in childhood results in generally mild or asymptomatic disease and can cause mononucleosis if acquired in adolescence. Following primary infection of epithelial cells and B cells, it establishes lifelong latency in memory B cells	[34,35,36,37,38]
HHV-8	Children: 2–6% HIV+ adults: 26–57% HIV- adults: 2–7%	Yes	Kaposi Sarcoma	Blood, saliva, sexual contact	Latent. During primary infection, HHV-8 infects different cell types such as B cells, monocytes, and endothelial cells. Following primary infection, lifelong latency is established mainly in B cells and endothelial cells	[39,40,41,42,43]
HSV-1	Children: 0–32% HIV+ adults: ~78% HIV- adults: 55–89%	Yes	Osteoporosis, cardiovascular events, dementia	Mostly oral–oral contact (oral herpes), perinatal	Latent. Primarily infects epithelial cells and neurons in the peripheral nervous system. In immunocompetent individuals, HSV-1 establishes lifelong latency in their sensory neurons while appearing phenotypically asymptomatic	[44,45,46,47]
HSV-2	Children: 0–16% HIV+ adults: ~55% HIV- adults: 20–28%	No	Osteoporosis, cardiovascular events, dementia	Sexual contact, perinatal; HSV-2 is associated with increased risk of HIV transmission	Latent. Like HSV-1, infects cells in the peripheral nervous system and establishes lifelong latency in the nucleus of sensory ganglia	[47,48,49]
HCV	Children: 0.2–0.4% HIV+ adults: ~18% HIV- adults: 0.8–1.0%	Yes	Liver disease	Primarily blood, vertical transmission	Chronic. Approximately 25% of those infected clear the virus spontaneously, while ~80% of people acutely infected will become chronically infected. And approximately 20% of those chronically infected will develop end-stage liver disease, hepatocellular carcinoma, or liver cirrhosis	[50,51,52]
HBV	Children: <0.001% HIV+ adults: 6–14% HIV- adults: <0.005–0.4%	Yes	Liver disease	Bodily fluids, blood, vertical transmission	Chronic. Most adults will go on to clear the virus spontaneously. Chronic infections can lead to severe liver damage resulting in cirrhosis or hepatocellular carcinoma	[53,54,55,56]

**Table 2:** Study design showing the distribution of individuals in the study. The target was n = 15 for each bin.

Age (Years)	Female		Male	
	HIV-	HIV+	HIV-	HIV+
0–10	15	15	15	13
10–20	15	15	15	15
20–30	15	15	11	13
30–40	15	15	10	8
40–50	15	15	14	10
50–60	15	15	14	11
60+	15	15	5	12

**Table 3:** Study sample demographics by sex and HIV status.

		Female			Male		
		HIV+ (n = 105)	HIV- (n = 105)	*p*-Value	HIV+ (n = 82)	HIV- (n = 84)	*p*-Value
Age (years), median (range)		38 (2–72)	37 (2–76)	0.92	31 (3–70)	31 (1–74)	0.37
Tobacco smoking, n (%)				0.15			0.38
	current	30 (29)	18 (17)		27 (33)	28 (33)	
	past	16 (15)	15 (14)		16 (20)	10 (12)	
	never	59 (56)	72 (69)		39 (48)	46 (55)	
Ethnicity, n (%)				0.16			
	White	44 (42)	53 (50)		29 (35)	36 (43)	0.57
	African/Black/Caribbean	36 (34)	23 (22)		24 (29)	20 (24)	
	Indigenous	19 (18)	18 (17)		15 (17)	11 (13)	
	Other	6 (6)	11 (10)		14 (17)	17 (20)	
Education attainment, n (%) (n = 268)				0.14			0.28
	Any university/college	42 (55)	58 (73)		19 (33)	30 (56)	
	High school—completed	11 (14)	8 (10)		17 (29)	11 (20)	
	High school—incomplete	15 (19)	9 (11)		11 (19)	12 (22)	
	Any grade school	2 (3)	0 (0)		1 (2)	1 (2)	
	Unknown	7 (9)	4 (5)		10 (17)	0 (0)	
	Household income, n (%) (n = 268)						
	<CAD 15,000 /year	32 (42)	27 (34)	0.31	17 (29)	21 (39)	0.68
	≥CAD 15,000 /year	38 (49)	49 (62)		34 (59)	33 (61)	
	Unknown	7 (9)	3 (4)		7 (12)	0 (0)	
Self-reported HCV status, n (%)		18 (17)	7 (7)	0.032	11 (13)	8 (10)	0.47
Self-reported HBV status, n (%)		7 (7)	1 (1)	0.035	4 (5)	0 (0)	0.057
HIV plasma viral load, <50 copies/mL (n = 187), n (%)		88 (84)			61 (74)		
CD4 count at visit, (cells/uL) (n = 168), median [IQR] (range)		610 [480–900] (50–1785)			545 [305–724] (5–1610)		

*p*-values indicate Mann–Whitney U, Chi-Squared tests, or Fishers Exact test depending on variable type. Household income and education attainment were available for adult participants only (>18 years old), n = 268.

**Table 4:** Study sample demographics by HIV status and sex.

		HIV+			HIV-		
		Female (n = 105)	Male (n = 82)	*p*-Value	Female (n = 105)	Male (n = 84)	*p*-Value
Age (years), median (range)		38 (2–72)	31 (3–70)	0.11	37 (2–76)	31 (1–74)	0.62
Tobacco smoking, n (%)				0.49			0.036
	current	30 (29)	27 (33)		18 (17)	28 (33)	
	past	16 (15)	16 (20)		15 (14)	10 (12)	
	never	59 (56)	39 (48)		72 (69)	46 (55)	
Ethnicity, n (%)				0.09			
	White	44 (42)	29 (35)		53 (50)	36 (43)	0.25
	African/Black/Caribbean	36 (34)	24 (29)		23 (22)	20 (24)	
	Indigenous	19 (18)	15 (17)		18 (17)	11 (13)	
	Other	6 (6)	14 (17)		11 (10)	17 (20)	
Education attainment, n (%) (n = 268)				0.049			0.062
	Any university/college	42 (55)	19 (33)		58 (73)	30 (56)	
	High school—completed	11 (14)	17 (29)		8 (10)	11 (20)	
	High school—incomplete	15 (19)	11 (19)		9 (11)	12 (22)	
	Any grade school	2 (3)	1 (2)		0 (0)	1 (2)	
	Unknown	7 (9)	10 (17)		4 (5)	0 (0)	
	Household income, n (%) (n = 268)						
	<CAD 15,000 /year	32 (42)	17 (29)		27 (34)	21 (39)	
	≥CAD 15,000 /year	38 (49)	34 (59)	0.19	49 (62)	33 (61)	0.50
	Unknown	7 (9)	7 (12)		4 (4)	0 (0)	
Self-reported HCV status, n (%)		18 (17)	11 (13)	0.55	7 (7)	8 (10)	0.59
Self-reported HBV status, n (%)		7 (7)	4 (5)	0.76	1 (1)	0 (0)	1.0
HIV plasma viral load <50 copies/mL, n (%) (n = 187)		88 (84)	61 (74)				
CD4 count at visit, (cells/uL), median [IQR] (range) (n = 168)		610 [480–900] (50–1785)	545 [305–724] (5–1610)				

*p*-values indicate Mann–Whitney U, Chi-Squared tests, or Fishers Exact test depending on variable type. Household income and education attainment were available for adult participants only (>18 years old), n = 268.

## Data Availability

Data are contained within the article and Supplementary Materials.

## Supplementary Materials

The following supporting information can be downloaded at https://www.mdpi.com/article/10.3390/v16050755/s1, Figure S1: Multivariable logistic regression modeling for total number of viral infections in (A) female participants and (B) male participants. Table S1: Concordance expressed as κ (95% CI) between plasma and serum biospecimens used in qualitative serological assays; Table S2: Region of birth of study participants; Table S3: Prevalence of seven chronic/latent viruses in sample; Table S4: All variables adjusted for in logistic regression model of total number of chronic/latent viruses; Table S5: All variables adjusted for in linear regression model of LTL.
