# Supplementary material for: Chronic and Latent Viral Infections and Leukocyte Telomere Length across the Lifespan of Female and Male Individuals Living with or without HIV
Source: Viruses. 2024 May 10;16(5):755. doi: 10.3390/v16050755 (PMC11125719; doi:10.3390/v16050755)
Supplement: Supplementary file 1 [file viruses-16-00755-s001.zip › viruses-2974673-supplementary.pdf]

Biospecimen handling, storage, and assay validation

For samples collected in Ontario or Quebec, they were shipped at room temperature within 24 hours. Following arrival at BC Women's Health Research (BCWH) lab the next day, they are frozen within 48 hours at -80°. Samples collected at the BCWH would not be processed until the next day to mimic conditions of the shipped samples. Samples in transit experience different handling conditions such as greater motion/shaking and temperature variability due to the nature of airplane transport. Despite these differences in conditions, our group has previously found that they had no significant effect on telomere length (Zanet 2013) [19].

Plasma or serum was thawed and prepared at UBC Hospital in tubes provided by the BC CDC and shipped in a secondary transport container on the same day to the BC CDC. Samples were assayed in an instrument using the same tubes used for transport to minimize handling and freeze-thawing. Both plasma and serum biospecimens were used. Validation experiments were conducted in order to verify the concordance between qualitative serological data in serum vs. plasma specimens from the same participant. We found no difference between using plasma and serum for the five serological assays use (Table S1).

Self-reported data

Between 2008 and 2013, HBV status was determined through asking the participants if they have ever had any liver complications. If yes, they would be asked to list the name, diagnosis date, and details of the condition. Those who reported a liver condition name of "Hep B", "Hep B Antibody+", or "Hep B carrier" (if prior to year 2000) where encoded as "positive" for HBV in this study. After 2013, the participants were asked "have you ever been exposed to Hep B?". Those who reported "Yes" to Hep B exposure via natural infection were encoded as "positive" with HBV in this study. HCV status was also self-reported and determined by asking the participant "do you have Hepatitis C?".

Table S1. Concordance expressed as κ (95% CI) between plasma and serum biospecimens used in qualitative serological assays.

Assay	n	% concordance	Cohen's kappa coefficient (κ)
CMV	28	92	0.76 ± 0.23 (0.32-1.00)
EBV	24	100	1
HSV-1	60	100	1
HSV-2	60	100	1
HHV-8	58	83	0.66 (0.46-0.85)

Table S2. Region of birth of study participants.

	**HIV+**			**HIV-**		
	**Female** (n=105)	**Male** (n=82)	**p-value**	**Female** (n=105)	**Male** (n=84)	**p-value**
**Region of birth**, n (%)						
North America	67 (64)	53 (65)		75 (71)	73 (87)	
Africa	24 (23)	16 (20)		4 (4)	0 (0)	
Asia	5 (5)	8 (10)	0.08	12 (11)	7 (8)	**0.03**
Europe	6 (6)	0 (0)		14 (13)	4 (5)	
South America	1 (1)	1 (1)		0 (0)	0 (0)	

Unknown	2 (2)	4 (5)		0 (0)	0 (0)	

Table S3. Prevalence of seven chronic/latent viruses in sample

	Female (n=210)	Male (n=166)	Δ (Delta)	HIV+ (n=187)	HIV- (n=189)	Δ	HIV+			HIV-			All participants (n=376)
							Female (n=105)	Male (n=82)	Δ	Female (n=105)	Male (n=84)	Δ	
**Virus, %**													
EBV	91	85	6	95	82	13	96	93	4	86	77	8	88
HSV-1	54	49	5	56	48	8	58	52	4	50	45	5	52
CMV	52	37	15	60	32	28	64	55	9	41	20	20	46
HSV-2	40	20	20	42	20	22	50	32	19	30	8	21	31
HHV-8	22	11	10	16	19	-3.5	17	13	4	27	10	17	17
HCV	12	11	0.5	16	8	8	17	13	4	7	10	-3	12
HBV	3.8	2.4	1.4	5.9	0.5	5.4	6.7	4.9	1.8	1.0	0.0	1.0	3.2

Table S4. All variables adjusted for in logistic regression model of total number of chronic/latent viruses

Variable	Effect size (95% CI)	P-value
**Female Sex (vs. male sex)**	0.45 (0.25—0.65)	P<0.0001
**HIV+ (vs. HIV-)**	0.49 (0.29—0.69)	P<0.0001
**Age (per 10 years)**	0.53 (0.40—0.66)	P<0.0001
**Ethnicity (vs. white)**		
African/Caribbean/Black	-0.11 (-0.57—0.35)	P=0.65
Indigenous	0.86 (0.41—1.32)	P=0.0002
Other	-0.28 (-0.84—0.27)	P=0.32
**Smoking (vs. never)**		
Current	0.52 (0.19—0.85)	P=0.002
Past	-0.09 (-0.45—0.28)	P=0.64
**Region of birth (vs. North America)**		
Africa	0.84 (0.22—1.47)	P=0.009
Asia	-0.20 (-0.87—0.47)	P=0.56
Europe	-0.29 (-0.93—0.35)	P=0.37

Table S5. All variables adjusted for in linear regression model of LTL

Variable	Effect size (95% CI)	P-value
**Female Sex (vs. male sex)**	0.24 (0.11—0.37)	P=0.0005
**HIV+ (vs. HIV-)**	-0.22 (-0.36— -0.09)	P=0.001
**Age (per 10 years)**	-0.42 (-0.51— -0.33)	P<0.0001
**Sex*HIV**	-0.13 (-0.26— -0.005)	P=0.042
**Ethnicity (vs. white)**		
African/Caribbean/Black	0.31 (0.06—0.55)	P=0.016
Indigenous	-0.14 (-0.43—0.14)	P=0.32
Other	0.13 (-0.17—0.42)	P=0.40
**Smoking (vs. never)**		
Current	-0.08 (-0.30—0.13)	P=0.44
Past	-0.01 (-0.25—0.24)	P=0.97
**Total # of non-HIV viruses (vs. 0-2)**		
3-4	-0.26 (-0.47— -0.04)	P=0.02
5-6	0.08 (-0.41—0.57)	P=0.75
**Virus Type**		

CMV	0.01 (-0.16—0.18)	P=0.92
EBV	-0.04 (-0.25—0.18)	P=0.74
HHV-8	0.19 (-0.003—0.39)	P=0.054
HSV-1	-0.07 (-0.23—0.10)	P=0.42
HSV-2	0.07 (-0.12—0.27)	P=0.46
HCV	-0.02 (-0.28—0.23)	P=0.85

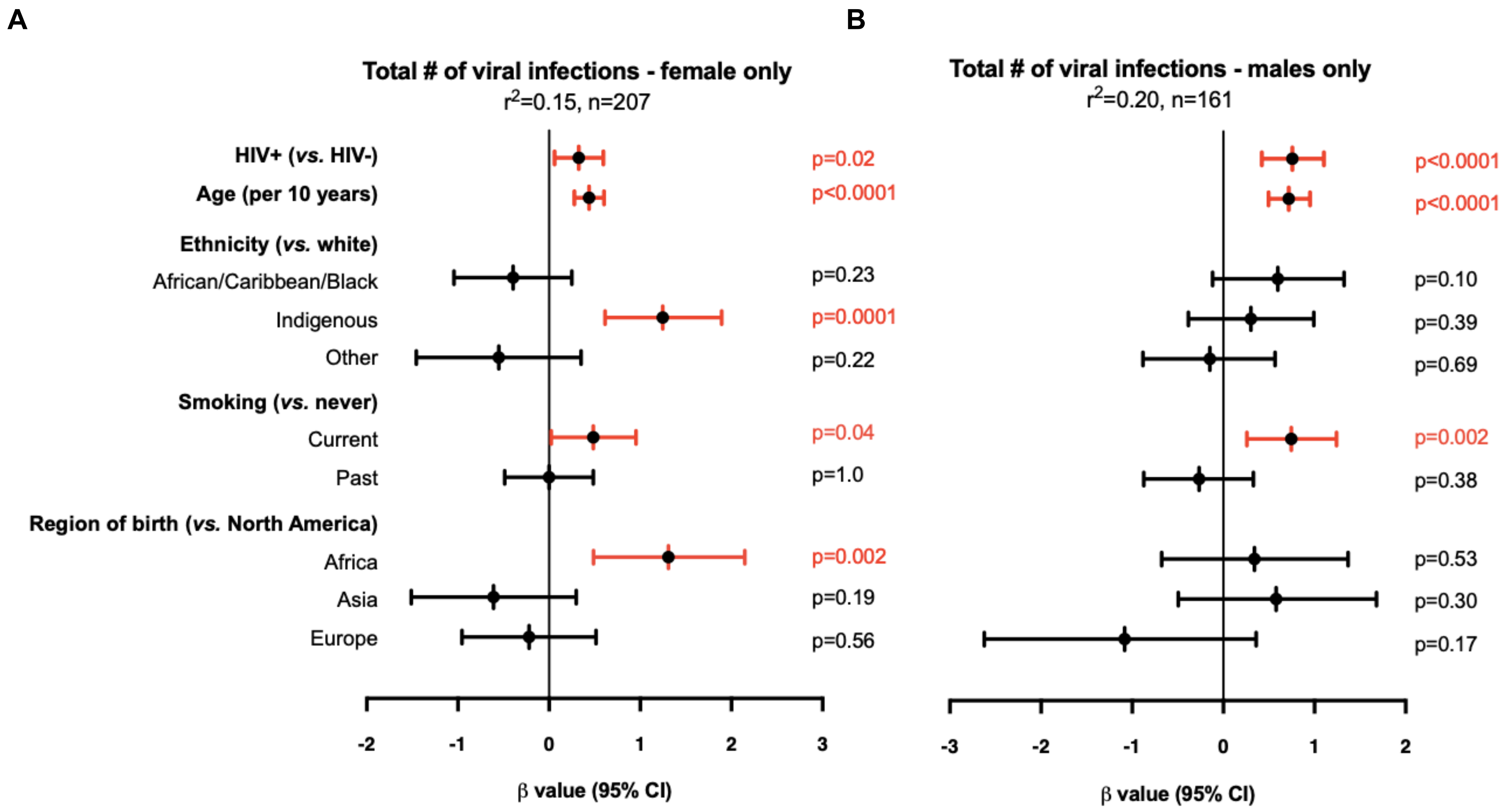
A
Total # of viral infections - female only
r2=0.15, n=207
HIV+ (vs. HIV-)
Age (per 10 years)
Ethnicity (vs. white)
African/Caribbean/Black
Indigenous
Other
Smoking (vs. never)
Current
Past
Region of birth (vs. North America)
Africa
Asia
Europe
p=0.02
p<0.0001
p=0.23
p=0.0001
p=0.22
p=0.04
p=1.0
p=0.002
p=0.19
p=0.56
-2
-1
0
1
2
3
β value (95% CI)
B
Total # of viral infections - males only
r2=0.20, n=161
p<0.0001
p<0.0001
p=0.10
p=0.39
p=0.69
p=0.002
p=0.38
p=0.53
p=0.30
p=0.17
-3
-2
-1
0
1
2
β value (95% CI)

Figure S1. Multivariable logistic regression modelling for total number of viral infections amongst (A) female participants and (B) male participants. The model shows that after adjustment for all variables shown, HIV+ status and older age remain independently associated with having more viral infections in both sex groups. Also, Indigenous ethnicity and being born in an African country are independently associated with having more infections amongst female participants only; current smoking status was associated with having more infections amongst both male and female participants. Female participants n=2 excluded for missing region of birth data, n=1 excluded for South America region of birth. Male participants n=4 excluded for missing region of birth data, n=1 excluded for South America region of birth.
